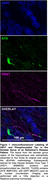# Decrowding Expansion Pathology for Visualization of Herpes Simplex Virus Type 1 and Tau Interaction in Alzheimer’s Disease

**DOI:** 10.1002/alz.095050

**Published:** 2025-01-09

**Authors:** Katherine Araya, Pablo Valdes, Giulio Taglialatela

**Affiliations:** ^1^ the University of Texas Medical Branch, Galveston, TX USA; ^2^ University of Texas Medical Branch, Galveston, TX USA

## Abstract

**Background:**

Chronically reactivating Herpes Simplex Virus Type 1 (**HSV1**) infection has been shown to produce key molecular and behavioral markers of Alzheimer’s disease (**AD**). Most notably, the accumulation of neurotoxic tau isoforms, neurofibrillary tangles (**NFTs**), and Amyloid‐beta plaques. Our study takes a unique approach to the systematic characterization of HSV1 biomarkers within the brain. We introduce the novel application of decrowding expansion pathology (**dExPath**) for immunofluorescent studies, a technique that sheds new light on the interactions between HSV1 proteins and tau. This innovative use of dExPath provides a visualization of HSV1 protein in the context of AD pathology, offering a fresh perspective on the hypothesized interactions between HSV1 proteins and Tau.

**Method:**

We aim to overcome the limitations of traditional imaging protocols, which are often hindered by spatial resolution and tissue density. To achieve this, we utilize dExPath, a technique that physically expands hippocampal tissue fourfold in an isotropic manner. This approach reveals disease biomarkers that are not visible with conventional staining techniques, allowing us to visualize HSV1 protein within AD clinical hippocampal specimens. Through this, we seek to establish a new understanding of the role of viral factors in the development of AD pathology.

**Result:**

**Our research has yielded a significant breakthrough**. We have successfully identified viral proteins within clinical AD hippocampal specimens using dExPath, a feat that was not possible with traditional immunofluorescent staining techniques.

**Conclusion:**

The findings of our research offer significant implications for the understanding of Alzheimer’s disease. By opening new avenues for therapeutic intervention targeting the interaction between HSV1 protein and tau, we provide potential insights into the disease’s etiology and potential treatment strategies. This could potentially lead to groundbreaking advancements in the treatment of Alzheimer’s disease.